# Supplementary material for: OsCSLD1 Mediates NH4+-Dependent Root Hair Growth Suppression and AMT1;2 Expression in Rice (Oryza sativa L.)
Source: Plants (Basel). 2022 Dec 19;11(24):3580. doi: 10.3390/plants11243580 (PMC9788582; doi:10.3390/plants11243580)
Supplement: Supplementary file 1 [file plants-11-03580-s001.zip › plants-1989005-supplementary.pdf]

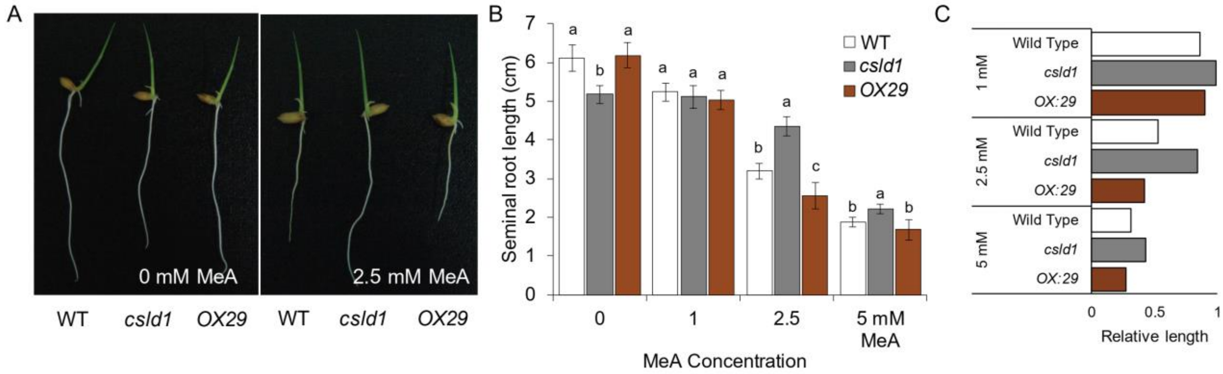

Supplementary Figure S1. *CSLD1:OX* lines are sensitive to Methylammonium (MeA). (a) Wild-type, *csld1* and *CSLD1:OX* seedlings grown in MeA free and 2.5 mM MeA treated seminal root lengths. (b) WT, *csld1* and *CSLD1:OX* seminal root lengths in 0 to 5 mM MeA. (c) Relative lengths of MeA treated seminal roots compared to non-treated root lengths. Scale bars = 1cm. line graph show mean values ± standard error of means (*n*=10).

Supplementary Table S1. Primers list used for qRT-PCR to quantify transcripts of *AMT1s*, and *OsCSLD*s transcript levels.

Primer	Sequence
UBQ1_F	AACCAGCTGAGGCCCAAGA
UBQ1_R	ACGATTGATTTAACCAGTCCATGA
AMT1:1_F	AGTACGTCGAGGAGATCTAC
AMT1:1_R	ACGTCGTTCGTTCTGGATTG
AMT1:2_F	TAGACATGGCCTCCCATCTC
AMT1:2_R	TAAGCATGATGTTCATGGTG
AMT1:3_F	AGGAGTACGTCGAGCTGATC
AMT1:3_R	CTTGCTCCGGCGACTTTCTG
OsCSLD1_F	GGAGCTAGGTGTTCGATCATG
OsCSLD1_R	AGTAGCTGATGAATCGCCCCGAG
OsCSLD2_F	GATCAGAAAGTGAGGCGATCG
OsCSLD2_R	GAGTAGGACATGCGCGAGAG
OsCSLD3_F	ATGCACGAGCACGAGAGATC
OsCSLD3_R	AGCGTCTGCTTCTTGCAGAG
OsCSLD4_F	GCTCACATGAAGGAGTGCTTG
OsCSLD4_R	CAAACAATCGCTTAATCACACC
